# *Borrelia turicatae* in Ticks from Animals in a Public Park, Aguascalientes, Mexico

**DOI:** 10.3201/eid3205.251925

**Published:** 2026-05

**Authors:** Edwin Vázquez-Guerrero, Gustavo Paniagua-Campos, Alexander R. Kneubehl, Paulina Estrada de los Santos, Job E. Lopez, J. Antonio Ibarra

**Affiliations:** Instituto Politécnico Nacional, Mexico City, Mexico (E. Vázquez-Guerrero, G. Paniagua-Campos, P. Estrada-de los Santos, J.A. Ibarra); Baylor College of Medicine, Houston, Texas, USA (A.R. Kneubehl, J.E. Lopez)

**Keywords:** Borrelia, Borrelia turicatae, bacteria, zoonoses, relapsing fever, opossum, Argasidae, feral fauna, Mexico

## Abstract

We obtained 5 isolates of *Borrelia turicatae* from ticks captured in a public park in Aguascalientes, Mexico. A serologic survey in resident fauna showed antibodies against *B. turicatae*. Relapsing fever borrelias are present in *Ornithodoros turicata* ticks and circulate in a zoonotic cycle, posing a risk for human infection.

*Ornithodoros turicata* ticks were originally described in Mexico by Alfredo Dugès at the end of the 19th Century. In 1936, it was implicated as the vector of tick-borne relapsing fever (TBRF) when febrile patients were first described in the city of Aguascalientes, in central Mexico (*1*). *Borrelia turicatae* is the species of TBRF spirochete transmitted by *O. turicata*, an argasid tick has been found in multiple regions of the United States and Mexico (*2*,*3*). We isolated *B. turicatae* from *O. turicata* ticks captured in the northern state of Sinaloa and used the diagnostic recombinant glycerophosphodiester phosphodiesterase (rGlpQ) antigen to detect circulating antibodies in clinical patients (*4*,*5*). Those findings indicated that *B. turicatae* and its vector are endemic in regions of Mexico, with spillover into human populations. However, TBRF in Mexico and in many other regions worldwide is a neglected disease that is often misdiagnosed because its symptoms are frequently confused with those of other diseases, such as malaria and brucellosis (*4*).

We obtained 5 isolates of *B. turicatae* from *O. turicata* ticks collected at La Pona Park in Aguascalientes, Mexico, in January and April 2023 (Figure 1). We examined tick specimens morphologically and molecularly by taxonomically analyzing a fragment of the mitochondrial genome, as described (*6*). Using a mouse model (either C57BL/6 or DBA/2J) (*5*), we evaluated the ticks by feeding them on the animals and assessing murine infection. We performed bacterial isolation as previously described by culturing blood samples from mice with spirochetemia in liquid, modified, and supplemented Barbour-Stoenner-Kelly II media supplemented with 10 µg/mL rifampin, 4 µg/mL phosphomycin, and 0.5 µg/mL amphotericin B (*5*). We amplified the 16S rRNA genes from each isolate by PCR, then sequenced and taxonomically analyzed them to confirm that all 5 isolates were *B. turicatae*; we named the isolates AGU1–AGU5. Of the 5 isolates, we sequenced genomic DNA from isolates AGU1–4 using NovaSeq X (Illumina, https://www.illumina.com); we used MinION for AGU1–3 and PromethION P2 Solo for AGU4 (Oxford Nanopore Technologies, https://nanoporetech.com). We base-called nanopore sequencing data using Dorado version 7.4.12 with the version 4.3.0 (https://github.com/nanoporetech/dorado) super-accurate base-calling model. We generated Illumina data using the Illumina DNA library prep kit 2 × 150 bp. We assembled chromosome-resolved and plasmid-resolved genome assemblies from Oxford Nanopore and Illumina data as previously described (*7*), with some modifications. We inferred a maximum-likelihood species tree as previously described; the tree demonstrated that AGU1–AGU4 clustered with *B. turicatae* 91E135, BTE5EL, and BTCAM1 strains (Appendix Figure). Both 91E135 and BTE5EL originated in Texas, USA, whereas BTCAM1 originated in Sinaloa, Mexico. AGU1–AGU5 are the southernmost *B. turicatae* isolates identified as of April 2026.

**Figure 1:**
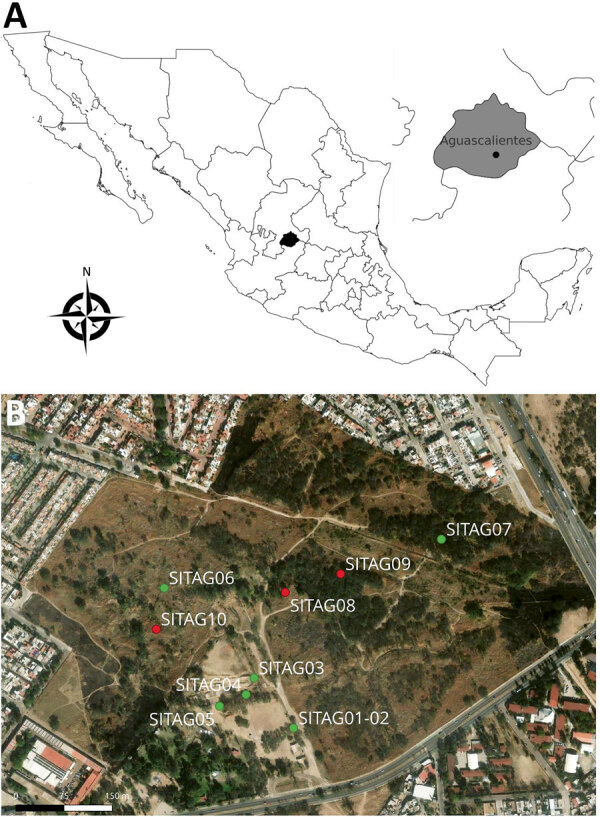
Locations of collection sites in study of *Borrelia turicatae* in ticks from animals in a public park, Aguascalientes, Mexico. A) Location of the state of Aguascalientes (black shading) in Mexico; inset at right shows the approximate location of the city of Aguascalientes (black dot). Map created using QGIS version 3.34 (https://qgis.org). B) Terrain map of La Pona Park in Aguascalientes. Green dots indicate collection sites where ticks were captured (SITAG01–07, 21°53′10.6” N 102°15′59.6” W; 21°53′13.4” N 102°16′01.7” W; 21°53′12.5” N 102°16′02.1” W; 21°53′18.2” N 102°16′06.6” W; 21°53′20.9” N 102°15′51.5” W; 21°53′11.1” N 102°16′03.1” W). Red dots indicate the 3 collection sites for resident fauna (SITAG08, 21°53′18” N, 102°16′00” W; SITAG09, 21°53′19” N, 102°15′57” W; SITAG10, 21°53′16” N, 102° 16′ 07” W). Map created using the ArcGIS imagery basemap (https://services.arcgisonline.com/ArcGIS/rest/services/World_Imagery/MapServer).

As part of our investigation, we captured wild fauna in La Pona public park to determine whether local animals were exposed to *Borrelia* spp. We collected blood samples in accordance with animal welfare guidelines (*8*). We captured 5 opossums (*Didelphis virginiana*), 1 deer mouse (*Peromyscus* sp.), and 1 feral cat (*Felis silvestris catus*) and tested serum samples by immunoblotting to detect antibodies against *B. turicatae* protein lysates, rGlpQ, and rBipA, as described previously (*4*,*5*). Results showed that the opossum and cat serum samples were reactive with *B. turicatae* protein lysates rGlpQ, and rBipA (Figure 2). None of the opossum samples were negative for the immunoblotting test, which might be a limitation of our study; however, opossum and cat serum samples from other states tested negative. Our results suggest that *B. turicatae* circulates in a tick–opossum–feral cat infectious cycle.

**Figure 2:**
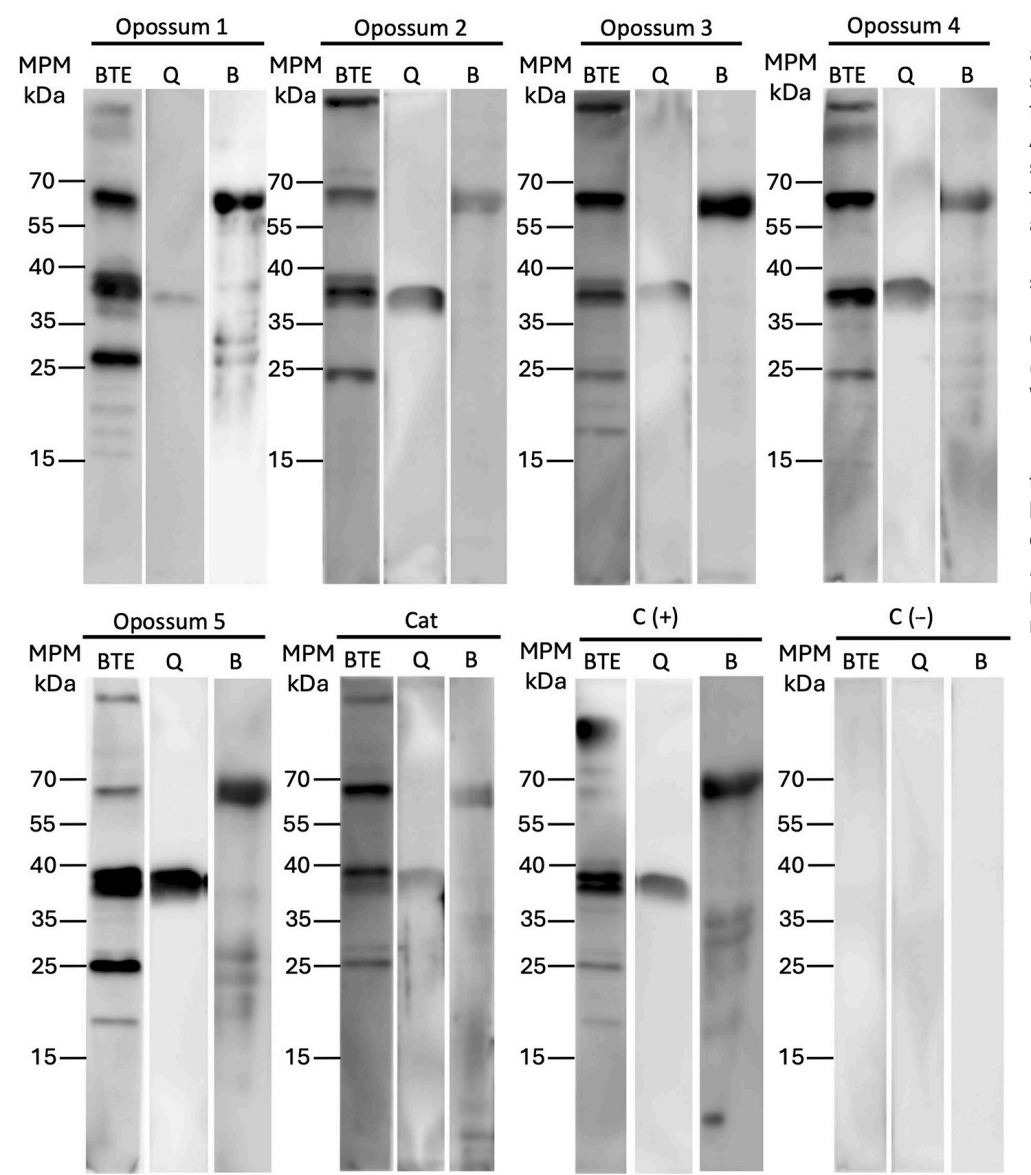
Immunoblot results assessing antibody responses in study of *Borrelia turicatae* in ticks from animals in a public park, Aguascalientes, Mexico. Images show responses in opossums and a feral cat captured in La Pona Park, along with mouse controls. C (+) is an immunoblot using a serum sample from a positive control mouse infected with *B. turicatae*. C (–) is from a negative control (serum from a nonexposed mouse). We used recombinant horseradish peroxidase protein A/G (Thermo Fisher Scientific, https://www.thermofisher.com) as the secondary ligand for the detection of both opossum and cat antibodies. BTE, *B. turicatae* protein extracts; Q, recombinant GlpQ; B, *B. turicatae* recombinant BipA.

Our findings support efforts to understand the mechanisms maintaining *B. turicatae* in nature, the distribution of infected *O. turicata* ticks, and the public health impact. Although we identified endemic foci of infected ticks, we did not determine the prevalence of *B. turicatae* in these populations. Understanding prevalence is relevant to public health because *B. turicatae* can be vertically transmitted from female ticks to their offspring at rates as high as 40% (*9*). Public parks and human dwellings have been shown to be a source for infected soft ticks in the southern United States and likely in human infections (*10*), but that relationship has been scarcely studied in Mexico. Given that unhoused and underserved persons reside in La Pona public park, they are at risk for exposure to *O. turicata* ticks and infection with *B. turicatae*. Our future work will focus on defining the tick–vertebrate infectious cycle of *B. turicatae* and assessing its effect on human populations living in or around La Pona Park. Clinicians should be aware of this potential zoonotic risk to humans in the area.

Appendix Additional information from study of *Borrelia turicatae* in ticks from animals in a public park, Aguascalientes, Mexico.
