# Supplementary material for: Borrelia turicatae in Ticks from Animals in a Public Park, Aguascalientes, Mexico
Source: Emerg Infect Dis. 2026 May;32(5):816–9. doi: 10.3201/eid3205.251925 (PMC13175112; doi:10.3201/eid3205.251925)
Supplement: Appendix — Additional information from study of Borrelia turicatae in ticks from animals in a public park, Aguascalientes, Mexico. [file 25-1925-Techapp-s1.pdf]

Article DOI: https://doi.org/10.3201/eid3205.251925

EID cannot ensure accessibility for supplementary materials supplied by authors. Readers who have difficulty accessing supplementary content should contact the authors for assistance.

Borrelia turicatae in Ticks from Animals in a Public Park, Aguascalientes, Mexico

Appendix

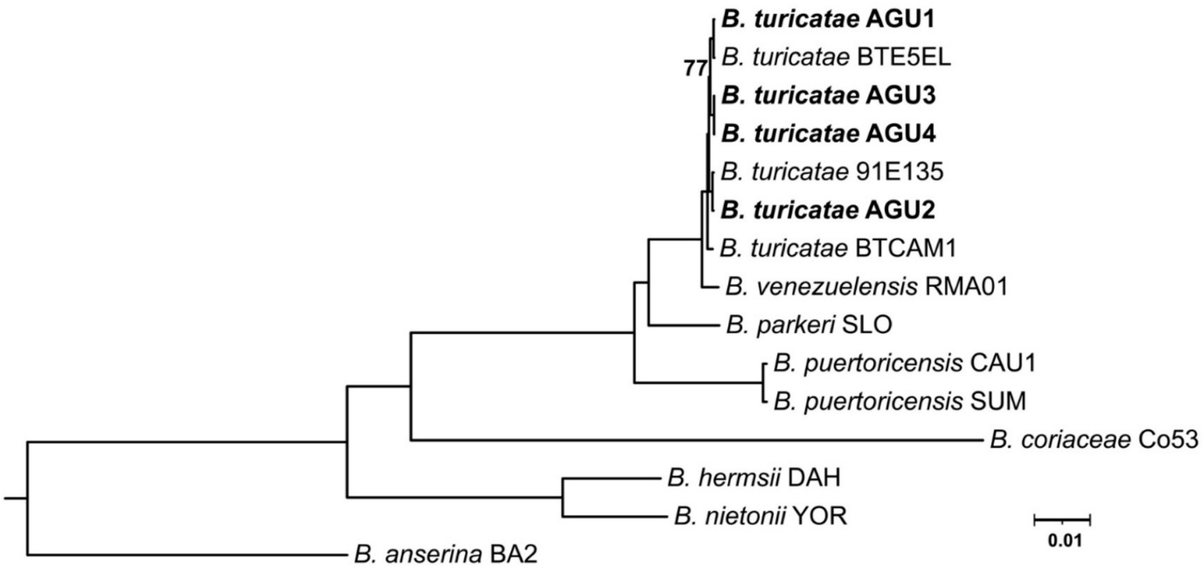

Appendix Figure. The isolates are shown in bold, and other relapsing fever *Borrelia* genomes were included in the analyses, and *Borrelia anserina* BA2 was used as the outgroup. The tree was generated with an edge-linked proportional partition model with 1,000 ultra-fast bootstraps replicates. Scale bar indicates 0.01 substitutions per site. GenBank RefSeq accessions for previously assembled genomes used for this analysis are: *B. anserina* BA2 (GCF_023035575.1), *B. hermsii* DAH (GCF_023035675.1), *B. nietonii* YOR (GCF_023035795.1), *B. coriaceae* Co53 (GCF_023035295.1), *B. puertoricensis* SUM (GCF_023035875.1), *B. puertoricensis* CAU1 (GCF_051132515.1), *B. parkeri* SLO (GCF_023035815.1), *B. venezuelensis* (GCF_023035835.1), *B. turicatae* 91E135 (GCF_023035855.1), *B. turicatae* BTE5EL (GCF_023035415.1), *B. turicatae* BTCAM1 (GCF_036362965.1). Phylogenomic analysis of *B. turicatae* isolates AGU1–4 was conducted as previously described (*7*), using panaroo v1.5.1 and IQ-TREE2 v2.3.6, and the results were visualized in iTOL v7.
